# Are Individual and Environmental Characteristics Associated With Running Performance in Female Runners of Different Age Categories?

**DOI:** 10.3389/fpsyg.2021.743744

**Published:** 2021-10-04

**Authors:** Mabliny Thuany, Ewa Malchrowicz-Mośko, Dariusz Kłoskowski, Thayse Natacha Gomes

**Affiliations:** ^1^Centre of Research, Education, Innovation and Intervention in Sport (CIFI2D), Faculty of Sport, University of Porto, Porto, Portugal; ^2^Faculty of Sport Sciences, Poznań University of Physical Education, Poznań, Poland; ^3^Koszalin University of Technology, Koszalin, Poland; ^4^Department of Physical Education, Federal University of Sergipe, São Cristóvão, Brazil

**Keywords:** running, predictors, female athletes, sports performance, ecological model

## Abstract

The purpose of this study was to identify the individual and environmental predictors associated with performance in female runners of different ages. The sample comprised 440 female Brazilian runners, who answered an online questionnaire, that provided information regarding height, weight, age (categories: “young adult”, “adult”, “early middle-age”, and “older adults”), socioeconomic status, and training characteristics (frequency and volume per week, running pace, race event, and running club participation). Information about environmental variables was obtained from the official institutes and comprised the human development index (HDI), athletics events, athletic tracks, and female homicide. A linear regression model, clustered by state and performed by age groups, was computed. The sample presented a mean running pace of 5:57min/km, and a mean BMI of 23.51kg/m^−2^. An increase in running pace and volume/week was observed with increasing age. In “young adults”, any of the variables were significantly associated with the performance. In “adult” group, only individual characteristics were statistically significantly related with the performance. In “early middle-age”, besides BMI (*β*=5.72; 95%CI=3.65–7.79) and training volume (*β*=−0.67; 95%CI=−1.07 − −0.27), the HDI was associated with the performance (*β*=−23.30; 95%CI=−44.11 − −2.49). In older adults, it was found an association between socioeconomic status (*β*=−19.47; 95%CI=−32.29 − −6.65), practice time (*β*=142.92; 95%CI=89.34–196.50), running event participation (*β*=−80.12; 95%CI=−114.35− −45.88), athletic events (*β*=33.44; 95%CI=15.16–51.72), and female homicide (*β*=−0.11; 95%CI=−0.17 − −0.05) with the performance, highlighting the influence of both individual and environmental characteristics. Information about the role of these constraints, and their relationships, in female runners’ performance, can be used to guide the development of projects/strategies aiming to increase their involvement in physical activities and sports practice, through the promotion of a more “friendly environment” to women, and providing support for decision-makers when suggesting/implementing public policies.

## Introduction

During the last years, it has been observed an increase in number of road running practitioners. Data covering 96% of the United States race events and 91% of the race results in Canada, Australia and a portion of Africa, Asia, and South America, during 1986 and 2018, showed an increase of 57.8% in the number of participants (Andersen, 2019). However, this increment in number of runners was not followed by increments in running performance, given the average time to conclude the events has increased during the last years (Vitti et al., 2019). For example, in 1986, the average finish time to conclude a marathon, considering results from non-professional runners, was 3h:52min:35s, whereas in 2018, the time was 4h:32min:49s – a slowdown of 40min:14s (RunRepeat, 2020a).

Aiming to understand these trends, researchers have paying attention on changes in runners’ profile (Breuer et al., 2013), and also on runners’ performance, trying to point out variables possibly related to this performance (Kozlovskaia et al., 2019; Thuany et al., 2020a). In this context, different anthropometric (Maciejczyk et al., 2014; Muñoz et al., 2020), physiological (Neves et al., 2019; Molinari et al., 2020), biomechanical (Willy and Paquette, 2019), psychosocial (Boullosa et al., 2020), training (Torres et al., 2020), and environmental variables have been investigated, regardless of age, distance, and/or the level of performance of these athletes (Muñoz et al., 2020; Thuany et al., 2020a). In general, studies show that most of the runners were male, with high socioeconomic level (Breuer et al., 2013), and that physiological indexes (VO_2máx_, anaerobic threshold and running economy) are important to running performance (Barnes and Kilding, 2015).

Since road running is an outdoor practice, practitioners are exposed to several constraints, derived from the natural, social, and built environments (Ren et al., 2020). In this context, researchers and official public organizations underline that built environment acts as an important factor to population sports practice involvement (Ettema, 2016), especially among women. Given the several barriers they are exposed (e.g., personal safety, lack of time to be engaged in leisure physical activity given double workday, difficult to access facilities due to urban security/violence, and male-dominated sports culture) (Sportscotland, 2008), different strategies have been proposed, planed by both government and society, to create safe and affordable venues/environments to encourage and promote the sports practice among women (e.g., running sisters clubs) (Running Sisters, 2019).

Taking into account information related to running, it is observed an increment in female participation in running events during the last decades (Andersen, 2019). Furthermore, female runners are more consistent at keeping their pace during the marathon race than their male peers (RunRepeat, 2020b). In addition, studies also show an increase in age of runners, meaning that road runners have been get older, with changes in mean age being observed between 1986 (35.2years) and 2018 (39.3years) (Andersen, 2019). However, most of the available data or studies conducted sampled male runners or elite female runners (Rust et al., 2011; Tawa and Louw, 2018; Noble and Chapman, 2020), with a gap of information related to non-professional female runners of different ages (Knechtle et al., 2011). Since the women involvement in running practice can provide them positive perceptions of mental and physical wellbeing (Kolhatkar and Shinde, 2020), as well as increments in their health status, it seems to be clear the relevance of giving more attention to this specific group.

Due to the relationship stablished between environment, as suggested by the ecological systems theory (Bronfenbrenner, 2011), running performance can be better understood taking into a count variables derived from different levels. But due to difficulties related to the access and analysis of data coming from these levels, which could provide deeply understanding about a given trait (in this case, running performance), the purpose of this study was not to test the applicability of the ecological theory to explain differences in running performance, but the theory was used as a theoretical approach/support to verify the association between individual- and states-level characteristic with female runner’s performance of different categories. Most of previous studies aiming to understand running predictors, paid attention to physiological, training, and morphological variables; however, it seems of relevance to consider that the training commitment can be associated with a plethora of environmental constraints. Information about the effect of these constraints, and their relationships, in the expression of female runners’ performance, can be used to guide the development of projects/strategies aiming to increase their participation in physical activities, to promote a more “friendly environment” to women, and to provide support for decision-makers when suggesting/implementing public policies. Therefore, the purpose of this study is to identify the individual and environmental predictors associated with performance in female runners of different ages.

## Materials and Methods

### Design and Participants

**Figure 1:**
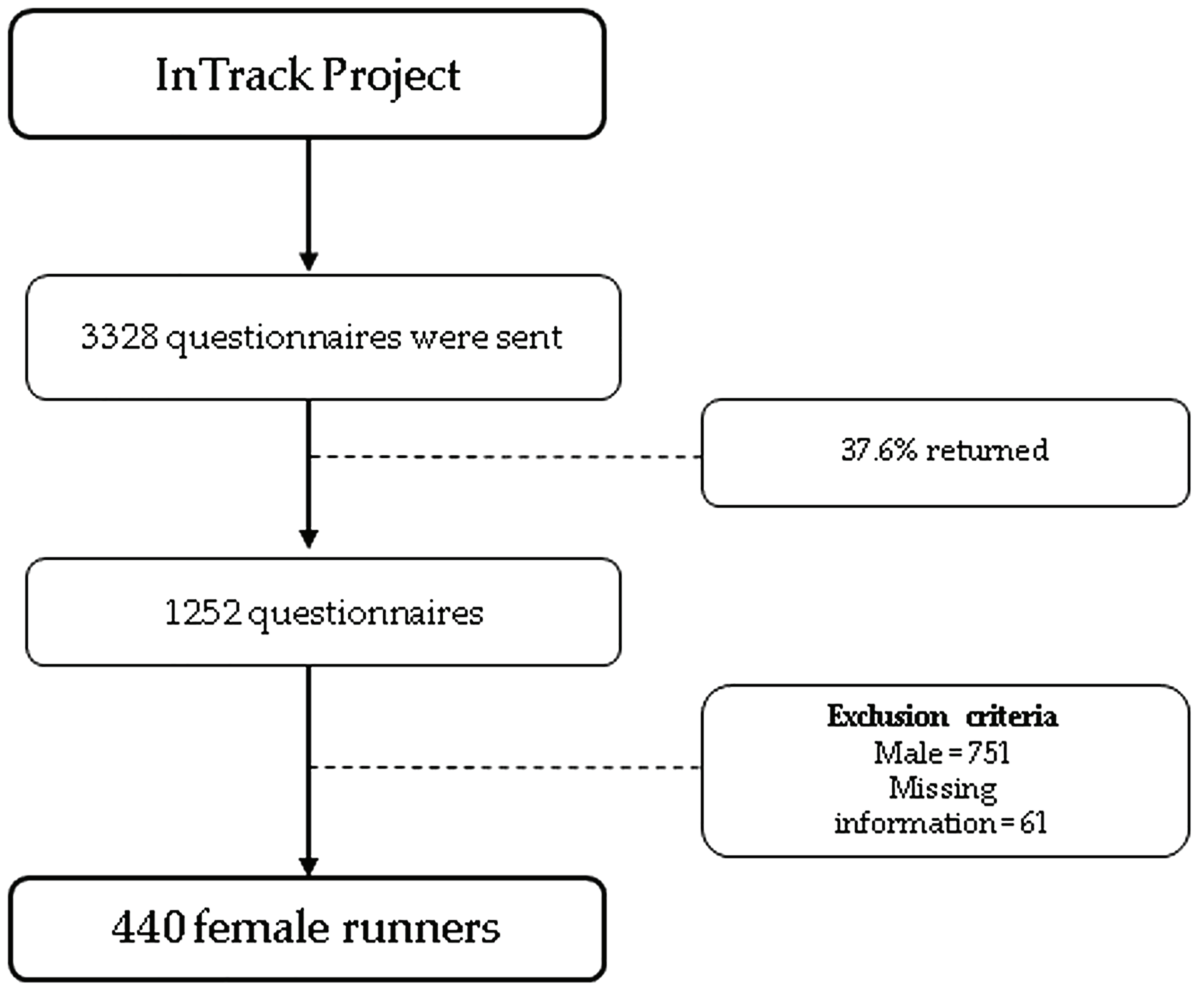
Flow chart of sampling strategy.

The present study is part of the InTrack Project,^1^ a cross-sectional research project developed to identify the runner’s predictors performance in non-professional runners. In the first approach, the project was conducted with 1,252 Brazilian non-professional runners of both sexes. Considering the inclusion criteria (participants self-classify as runner and answer the online questionnaire) and exclusion criteria (not answer/provide a non-sense answer to mandatory questions, aged under 18 years, male), the sample for the present study comprised 440 female runners, aged between 19 and 65years old (Figure 1), from all the five Brazilian regions (Southeast=152; South=62; North=39; Midwest=40; and Northeast=147). This study was approved by the Ethical Committee of Federal University of Sergipe (protocol n° 3.558.630). Requirement for informed consent was obtained by the participants, and all runners were informed about procedures and purposes of the study.

### Instruments

The information was collected through the online questionnaire “Profile characterization and associated factors for runner’s performance” (Thuany et al., 2020b), which was available to runners answers between September/2019 and March/2020. The instrument was available in social media (through groups and clubs) and allowed obtaining information related to: (1) anthropometric characteristics, (2) sociodemographic aspects, and (3) training information.

#### Individual Characteristics

##### Anthropometric Characteristics

Height (cm) and body mass (kg) were self-reported, as performed in previous studies (Nikolaidis and Knechtle, 2020). The body mass index (BMI) was computed through the standardized formula [body mass (kg)/height (m)^2^].

##### Age Categories

Based on runners’ age, they were categorized in different groups, namely, “young adult” (18–29years); “adult” (30–39years); “early middle-age” (40–49); and “older adults” (≥50years). Age category was thought based in a previous study conducted in two of the main Brazilian race events (Thuany et al., 2021a).

##### Socioeconomic Status

Runners were asked to provide an estimate of their monthly income, in a Likert scale format, based on Brazilian minimum wage in 2019 (República Federativa do Brasil, 2019). Furthermore, answers were organized in the following categories: a) Low ≤ R$998.00 (≤241.06US$), b) R$998.00<Medium ≤ R$2994.00 (or about 241.06US$<Medium ≤723.18US$); c) R$2994.00< Medium-high ≤ R$4990.00 (or about 723.18US$<Medium-high ≤1.205US$); and d) High >R$4990.00 (or about >1.205US$).

#### Training Characteristics

##### Running Pace

The time spent to cover 1km was considered as dependent variable in the present study. Participants were asked to enter information regarding the running pace (min/km) in their preferred distance. To data analysis, the running pace was used in seconds/km (s/km).

##### Practice Time

Runners were categorized in two groups, namely, “until 1year” of practice or “more than 1 year” of practice.

##### Training Frequency/Week

It was reported in counts (1–7 train/week). Based in previous studies that showed most of the recreational runners train at least three times/week (Fonseca et al., 2019; Thuany et al., 2020a), runners were split in the following groups: “at last 3 sessions/week” or “more than 3 sessions/week.”

##### Training Volume/Week

The mean weekly value was reported (in kilometers) by runners.

##### Race Events

Information about participation in any race event in the last 12months (“yes” or “no”) was provided by participants.

##### Running Club Participation

Information regarding the participation in any running club was provided (“yes” or “no”).

#### Environmental Characteristics

##### Human Developed Index

The Human Developed Index (HDI) considers in long-term three basic dimension by human development: income, health, and education. Based on the United Nations Development Program [Programa das Nações Unidas (PNUD), 2020], information was obtained and states were categorized as “medium (≤0.699)”, “high (≥0.700 and≤0.799)”, or “very high (≥0.800)” HDI (none of the states presented an HDI classified as low (<0.600)).

##### Athletic Events

Information about the existence of athletics events in non-professional venue, per state, was obtained, based on the Basic State Information Research from the Brazilian Institute of Geography and Statistics (Instituto Brasileiro de Geografia e Estatística, 2016). The variable was categorized in “yes (there is)” or “no (there is not).”

##### Athletic Tracks

The number of athletic tracks in states was obtained from the Basic State Information Research from the Brazilian Institute of Geography and Statistics (Instituto Brasileiro de Geografia e Estatística, 2016), considering the year of 2016.

##### Female Homicide

Considering barriers to woman physical activity and the influence of environment to training (Allen-Collinson, 2008), woman homicide was used as the violence index indicator. It expressed the total number of female homicides, per state, in 2017 (Cerqueira et al., 2019).

### Data Analysis

Descriptive information was presented in mean (standard error), range (min – max), and frequency (%). Normality was tested by the Kolmogorov–Smirnov test, considering age categories. Adult and early middle-age groups presented non-parametric distribution for running pace. However, for all groups, standardized residual presented normality and mean zero. Homoscedasticity was tested and based in White’s test, only in the adult group, this assumption was violated. So, the Kruskal-Wallis test was performed to estimate differences between age groups in continuous variables. Since women are from different states, we performed a linear regression model, clustered by state, and performed by age groups (young adults, adults, early middle-age, and older adults) to estimate the predictors associated with running performance. All predictors were considered in a unique model, split into: individual characteristics (BMI, SES, training frequency (“until 3 train/week”; “>3 train/week”), training volume, practice time (“until 1year”; “> 1year”), running club (“no”; “yes”), running event (“no”; “yes”)) and states variables (HDI (“medium”, “high”, and “very high”), athletics events (“no”; “yes”), athletics track, and female homicides). All analyses were undertaken in the STATA V.14.0 software (STATA Corp, College Station, Texas, United States), with a 95% of confidence level.

## Results

The sample presented a mean running pace of 5:57min/km, and a mean BMI of 23.51kg/m^−2^. Descriptive information, by age groups, is presented in Table 1. An increase in running pace and volume/week was observed with increasing age. Most of the female runners reported a training frequency until three train/week, except for those aged >50years, and about half of runners indicated a training frequency>3 train/week. For all groups, most of the subjects reported to take part in running club and were enrolled in running event. Information regarding Brazilian states showed that the majority of the states was classified as having medium or high HDI. In almost all states, there were athletics events, and the number of athletic tracks ranged between 0 and 8. A mean of 182.81 female homicides/year was observed.

**Table 1:** Descriptive information of runners, by age group (mean (standard error) and frequency (%)), and states-level characteristics.

Female runners’ characteristics	Young adults (*n*=64)	Adults (*n*=177)	Early middle age (*n*=116)	Older adults (*n*=31)	
					Kruskal-Wallis test
Running pace (s/km)	347.51 (6.72)	351.22 (3.85)	368.57 (4.39)	379.06 (8.62)	*p* <0.001
Body Mass Index (kg.m^−2^)	22.8 (0.33)	23.34 (0.22)	24.36 (0.28)	23.87 (2.97)	*p* =0.004
Training volume (km/week)	25.04 (2.17)	26.44 (1.31)	28.67 (1.6)	29.53 (12.3)	*p* =0.367
**Economic status**
Low	6 (9.52%)	6 (3.43%)	1 (0.88%)	1 (3.23%)	
Medium	44 (69.84%)	88 (50.29%)	35 (30.70%)	9 (29.03%)	
Medium-high	13 (20.63%)	81 (46.29%)	76 (66.67%)	21 (67.74%)	
High	0 (0%)	0 (0%)	2 (1.75%)	0 (0%)	
**Training frequency**
Until 3 train/week	49 (76.56%)	122 (68.93%)	82 (70.69%)	15 (48.39%)	
>3 train/week	15 (23.44%)	55 (31.07%)	34 (29.31%)	16 (51.61%)	
**Practice time**
Until 1year	19 (29.69%)	38 (21.59%)	11 (9.48%)	1 (3.23%)	
>1year	45 (70.31%)	138 (78.41%)	105 (90.52%)	30 (96.77%)	
**Running club**
No	18 (28.57%)	37 (20.90%)	15 (12.93%)	3 (4.11%)	
Yes	45 (71.43%)	140 (79.10%)	101 (87.07%)	28 (8.92%)	
**Running event**
No	11 (17.19%)	12 (6.78%)	5 (4.31%)	1 (3.23%)	
Yes	53 (82.81%)	165 (93.22%)	111 (95.69%)	30 (96.77%)	
**States-level characteristics**
**HDI**
Medium	14 (51.9%)				
High	12 (44.4%)				
Highest	1 (3.7%)				
**Athletic events**
No	5 (18.5%)				
Yes	22 (81.5%)				
**Athletic track**	0–8	
**Female homicides (year)**	182.81 (149.15)	

**Figure 2:**
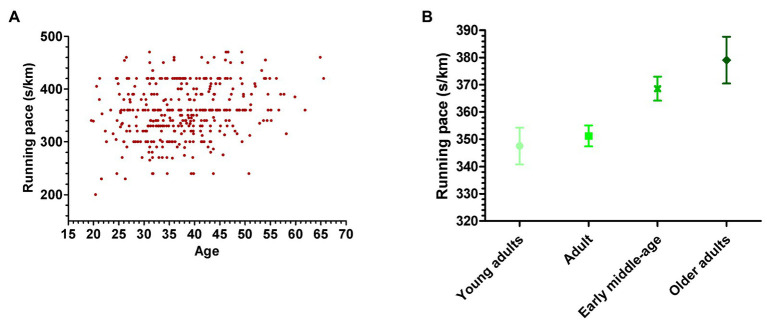
Running pace (s/km) for each subject **(A)** and box-plot to running pace by age groups **(B)**.

Figure 2 presents values for running pace according to age (Figure 2A) and by age-groups interval (Figure 2B). It is possible to observe a variability in pace among female runners, with an increase in time to conclude 1kilometer with increasing age.

Linear regression results are presented in Table 2. For “young adults”, the variables included in the model did not present an association with running pace. On the other hand, among female runners from the “adults” group, the predictors explained 39% of the performance variance. An increase in BMI was associated with a decrease in performance, but runners with more than 1year of practice time and>3 train/week presented better pace values, in approximately 15s/km and 12s/km, respectively, when compared against their peers. For the “early middle-age” group, similar result for BMI was observed (increasing in BMI was associated with decreases in the performance). A higher training volume presented a direct relationship with the performance (*β*=−0.67; 95%CI=−1.07-–0.27), and increases in HDI of the state where runners live, implies in an increase in performance of about 23s/km (95%CI=−44.11-–2.49). The built model explained 34% of the performance variance. Among “older adults” group, the built model explained 73% of the performance variance in the studied group. Besides the role of training variable in running pace (practice time: *β*=142.92; 95%CI=89.34-–196.50), increases in Socioeconomic Status (SES) indicated a reduction in running pace (*β*=−19.47; 95%CI=−32.29-–196.50). In this group, runners who reported to take part in a running event in the last 12months presented a better running pace than their peers in more than 1min/km (*β*=−80.12; 95%CI=−114.35-–45.88). Furthermore, athletics events (*β*=33.44; 95%CI=15.16-–51.72) and female homicides (*β*=−0.11; 95%CI=−0.17-–0.05) were also associated with the performance.

**Table 2:** Results to linear regression clustered by state, considering age group.

Variables	Young adults			Adults			Early middle age			Older adults		
	β	95%CI		β	95%CI		β	95%CI		β	95%CI	
Constant	288.18	0.00	132.42	213.06	157.19	268.93	281.08	222.40	339.76	202.91	−15.01	420.83
BMI (kg/m^2^)	3.61	−1.76	8.97	7.36^*^	4.83	9.90	5.72^*^	3.65	7.79	6.75	−0.09	13.60
SES	16.47	0.33	−17.95	3.48	−5.77	12.72	4.08	−12.92	21.09	−19.47^*^	−32.29	−6.65
Practice time (>1year)	−15.21	0.15	−36.31	−15.40^*^	−26.44	−4.35	−25.10	−61.10	10.90	142.92^*^	89.34	196.50
Frequency (>3 train/week)	−4.17	0.83	−43.50	−12.00^*^	−23.24	−0.76	−2.09	−19.52	15.33	−0.51	−32.89	31.87
Volume/week	−0.99	0.23	−2.64	−0.47	−1.05	0.10	−0.67^*^	−1.07	−0.27	0.00	−1.04	1.03
Running event (yes)	−13.36	0.52	−56.24	4.00	−26.42	34.42	4.85	−36.74	46.45	−80.12^*^	−114.35	−45.88
Running group (yes)	20.47	0.30	−20.07	8.57	−7.60	24.74	−15.34	−39.64	8.95	−22.83	−67.32	21.67
HDI	33.25	0.07	−2.67	−11.54	−33.31	10.22	−23.30^*^	−44.11	−2.49	5.86	−9.31	21.03
Athletic events (yes)	−20.73	0.08	−44.27	−18.58	−40.10	2.94	−3.28	−19.00	12.44	33.44^*^	15.16	51.72
Athletic Track	6.00	0.52	−13.36	4.05	−1.90	10.00	4.75	−3.91	13.41	4.90	−1.07	10.87
Female homicides	−0.09	0.16	−0.23	−0.02	−0.08	0.04	0.02	−0.02	0.07	−0.11^*^	−0.17	−0.05
R^2^	0.34			0.39			0.34			0.73		

BMI – kg.m^−2^;

* indicates significate value; and 95%CI – confidence interval.

## Discussion

Increase of female involvement in running events has been observed in last years (Andersen, 2019). Understanding the role of individual and environmental characteristics in the expression of the performance is a relevant key in the development of the long-term training and to implement projects and approaches aiming to promote and develop a friendlier environment. Therefore, the main purpose of this study was to identify the individual and environmental predictors associated with performance in female runners at different ages. Based on the Bronfenbrenner’s theory (Urie Bronfenbrenner, 1977; Bronfenbrenner, 2011), it was considered variables derived from different levels. The main findings were that (1) there was a variability in performance among female runners, with a decrease in the performance with increasing age, and (2) there were differences in variables significantly associated with running performance according to age groups.

Increment in age was associated with decreases in performance, with a mean difference of about 32s between “young adults” and “older adults” groups, meaning that runners from this last group were slower than those from the first group. This difference (32s) represents a difference of ≈22min in the time to conclude a marathon race. Most endurance runners experience a reduction in performance with age (Zavorsky et al., 2017; Waldvogel et al., 2019), and this change in performance is associated with behavioral, biomechanical, and physiological factors (Dahl et al., 2020). For example, changes in lifestyle (e.g., reduction in training volume and frequency; changes in nutritional habits) can lead to increment in body fat or reduction in cardiovascular function, with consequences in running performance (Willy and Paquette, 2019). Previous studies reported that body fat and training characteristics were also associated with race time among master half-marathoners, master marathoners, and master ultra-marathoners (Knechtle et al., 2012). Additionally, the natural aging process leads to an overall decline in central and peripheral factors associated with physiological indicators. For example, the aerobic capacity (VO_2máx_), an important physiological factor for running performance, is a product of cardiac output and arteriovenous oxygen differences, and these factors also reduce with increasing age (Fuziki, 2012; Willy and Paquette, 2019). These aspects can explain the results of the present study, where older adults presented the lowest running pace, but they also reported the highest values of volume/week and a training frequency higher than three train/week in comparison with other groups.

For the “young adults” group, the built model was not associated with running performance. From these results, it is important to take into account other individual (e.g., body fat percentage, running motivation, working time, perception about the environment to physical activity, and company for physical activity) and environmental characteristics that can explain differences in the performance. On the other hand, for “adults” group, it was observed a significant predictor role of anthropometric and training characteristics in the performance. Regarding the BMI, previous studies indicated an inverse relationship between this health indicator and running performance (Sedeaud et al., 2014; Mooses and Hackney, 2017; Thuany et al., 2020a). Given running is a “sensitive weight sport” (Silva, 2019), body mass is a key component of energy expenditure in running, making heavier subjects spend more energy to run (Sedeaud et al., 2014). Besides that, additional body weight can represent a critical mechanical load rise to the leg swing movement. Moreover, about the practice time and training frequency, training exposure may lead to increments in the performance due to changes in aerobic capacity, lactate threshold, and running economy (Fuziki, 2012; Souza et al., 2014), and these factors are usually associated with running performance.

It is interesting to note that in “early middle-age” and “older adults” groups, besides the individual and training characteristics – namely, anthropometric, socioeconomic, and training variables – social and environmental variables were also related to performance. For the “early middle-age” group, a higher HDI state was associated with a better running performance, meaning that female runners living in states with higher HDI spent about 23s less to cover 1km. In a study with Brazilian swimming athletes (Gomes-Sentone et al., 2019), it was observed that HDI, income, and educational level were relevant social indicators for athletes performance. Similar results were reported among Brazilian soccer players (Costa et al., 2013), where young athletes born in cities with medium or higher HDI presented more chances to be successfully in comparison with their peers from low HDI states. Furthermore, among Brazilian professional endurance runners (Thuany et al., 2021c), it was identified that the best athletes were from the Southeast and South regions, which are the regions whose states show the highest HDI and the best socioeconomic indicators at a national level. Association between HDI and sports practice/performance highlights a more favorable environment to sports development (e.g., infrastructure, sports programs, coach development program, and economic condition) (Cote et al., 2006). Specifically in non-professional runners, these results can be associated with the fact that these states can provide more accessible places, and a urban plan that promotes the mobility and also looks like more safety for woman, contributing to a decrease in gender inequity (World Bank Group, 2015). Therefore, it is possible that female runners living in these places dispose for a more friendly environment for training, and this training commitment can be expressed in a better running performance (Thornton and Scott, 1995). Moreover, HDI derives from “education,” “income,” and “health” indicators, what can suggest that higher HDI can provide more information to citizens about the relevance to be physically activity. Previous studies indicated a positive association between HDI and leisure physical activity (Rodrigues et al., 2017).

Among the “older adults” group, understanding the role of the environment in the performance of these women is of relevance due to several constraints (e.g., social, accessibility, economic factors, poor health, and lack of companions with whom to participate) observed in this age range (Moschny et al., 2011; Yu et al., 2020). Regarding the effect of the built environment in the leisure time physical activity, Yu et al. (2020) showed that older women were more easily affected by the built environment than men and that factors as residential density, street connectivity, and crime safety were pointed as important barriers to woman physical activity. In the present study, it was found that besides sociodemographic aspects and practice time, social indicators were also associated with the performance. In this context, women that reported take part in a running event in the last 12months presented a better performance, what can be associated with a more motivation for running practice and more training commitment (frequency and volume/week). Regarding states variables, it was observed that runners living in states with athletics events spend more time to cover 1km, what goes in opposite of results from previous studies that highlight the significant role of the presence of sports events in the performance of athletes/practitioners (Thuany et al., 2021b). However, it is interesting to note that these events investigated in the present study do not indicate only running events, or even the involvement of older adults. Moreover, an inverse relationship was observed between performance and female homicide, notwithstanding its effect on the performance seems to be small. The inclusion of this variable in the model is related with the fact that violence is one of the main factors that impair women to engage in outdoor activities.

Despite all efforts, this study is not free of limitations. Firstly, when sample was categorized into age groups, differences in sample size was observed, with special attention in the “older adults” group, and this may limit the generalization of results. Secondly, self-reported information could be prone to misleading data, notwithstanding the previously validation of the questionnaire, and the fact that this strategy has been largely used in the literature (Vickers and Vertosick, 2016; Thuany et al., 2020b). Thirdly, environmental information was obtained from secondary data, what can be difficult to be updated. Furthermore, environmental-related variables were obtained based on runners’ state of residence, and not based on their cities of residence. Additionally, we did not differentiate runners based on categories for non-professional runners (e.g., novice, amateur, and recreational), what can bias the results related to the identification of performance predictors, since they may not be the same if categories were considered; but taking into account the sample size, splitting sample in age categories and also in runners performance groups would impair during statistical analysis. For this reason, we only considered the age groups. However, to the best of our knowledge, this is the first country-based study that analyzed running predictors in woman at different age groups. Information can be used to guide future physical activities intervention for women and can be used to help in long-term training. Given that, practical implication can be organized in different levels, such as political and organizational changes aiming to promote more running events, which should be accessible and attractive for female participation. Since running events participation can be a motivational factor to women commitment in running training, it could provide better performance as well as the practice maintenance for long time, with benefits for health outcomes. At micro-level, the coaches and sports club can incentive women to get involve in competition and also their practice maintenance.

## Conclusion

Different variables were associated with women running performance by age groups. In “adults”, it was found that anthropometric and training characteristics (BMI, practice time, and training frequency) can be used to differentiate performance among women. In “early middle-age” group, it was also observed the role of BMI and the training characteristics (it was found that volume/week was associated with the performance). In addition, in this group, HDI was also associated with running performance. For the oldest group, SES, practice time, participation in running event, athletics events, and female homicide in the place where runners live were significantly associated with performance. Especially for the oldest group, it is suggested that social environment should be deeply explored in future studies, and strategies must be thought and developed with the purpose to provide a safer and friendlier environment to women physical activity and sport practice.

## Data Availability Statement

The datasets analyzed in this article are not publicly available. Requests to access the datasets should be directed to correspondence author.

## Ethics Statement

The studies involving human participants were reviewed and approved by Ethical committee of Federal University of Sergipe. The patients/participants provided their written informed consent to participate in this study.

## Author Contributions

MT and TNG: conceptualization, methodology, and writing – original draft preparation. MT: formal analysis. MT, TNG, EM-M, and DK: writing – review and editing. All authors have read and agreed to the final version of the manuscript.

## Conflict of Interest

The authors declare that the research was conducted in the absence of any commercial or financial relationships that could be construed as a potential conflict of interest.

## Publisher’s Note

All claims expressed in this article are solely those of the authors and do not necessarily represent those of their affiliated organizations, or those of the publisher, the editors and the reviewers. Any product that may be evaluated in this article, or claim that may be made by its manufacturer, is not guaranteed or endorsed by the publisher.

## Footnotes

^1^ https://intrackproject.wixsite.com/website
